# Three New Highly Oxygenated Germacranolides from *Carpesium Divaricatum* and Their Cytotoxic Activity

**DOI:** 10.3390/molecules23051078

**Published:** 2018-05-03

**Authors:** Tao Zhang, Jin-Guang Si, Qiu-Bo Zhang, Jia-Huan Chen, Gang Ding, Hong-Wu Zhang, Hong-Mei Jia, Zhong-Mei Zou

**Affiliations:** 1 Institute of Medicinal Plant Development, Peking Union Medical College and Chinese Academy of Medical Sciences, Beijing 100193, China; zt830423@163.com (T.Z.); sjgking@126.com (J.-G.S.); zhangqiubolsh@163.com (Q.-B.Z.); chenjiahuan0107@163.com (J.-H.C.); gding@implad.ac.cn (G.D.); 18101318775@163.com (H.-W.Z.); rainbow-grape@163.com (H.-M.J.); 2 School of Traditional Chinese Medicine, Shenyang Pharmaceutical University, Shenyang 110016, China

**Keywords:** *Carpesium divaricatum*, germacranolides, absolute configuration, cytotoxicity

## Abstract

Three new highly oxygenated (**2**–**4**), and two known (**1** and **5**) germacranolides, were isolated from the whole plant of *Carpesium divaricatum*. The planar structures and relative configurations of the new compounds were determined by detailed spectroscopic analysis. The absolute configuration of **1** was established using the circular dichroism (CD) method and X-ray diffraction, and the stereochemistry of the new compounds **2**–**4** were determined using similar CD spectra with **1**. The new compound **2** and the known compound **5** exhibited potent cytotoxicity against hepatocellular cancer (Hep G2) and human cervical cancer (HeLa) cells, superior to those of the positive control *cis*-platin.

## 1. Introduction

The genus *Carpesium* (Asteraceae) includes 25 species worldwide, most of which are distributed across Asia and Europe, particularly in southwest China [1,2]. In China, Korea, and Japan, many *Carpesium* species have been used for the treatment of fevers, colds, bruises, and snake bites, due to their antipyretic, analgesic, vermifugic, hemostatic, detoxifying, and anti-inflammatory properties [2]. The genus is rich in diverse sesquiterpenoid lactones, such as eudesmanolides and germacranolides [2,3,4,5,6]. Previous investigations indicate that sesquiterpenoid lactones possessing an *α*-methylene-*γ*-lactone moiety are cytotoxic to human cancer cells [2,3,4,5,6,7]. Recently, six sesquiterpenoid lactones with new skeletons displaying significantly cytotoxic activity were isolated from *Carpesium* plants [8,9].

*Carpesium divaricatum* Sieb.et Zucc is widely distributed in China, and is traditionally used for the treatment of fevers, colds, bruises, insect bites and inflammatory diseases [10,11,12,13,14,15]. Previous investigations of this plant reported the isolation of germacrane-type sesquiterpene lactones [4,14,15,16,17]. The parent nucleus of the germacranes contains a 10-membered ring with different post-modifications to produce diverse structural features. Many skeletal types of germacranolides with broad biological activities, such as cytotoxic and anti-inflammatory properties, have been isolated from the genera *Carpesium*, *Inula* and *Allagopappus* [5,6,11,15,16,17,18,19,20,21,22,23,24,25,26]. Our previous study led to the isolation, structural elucidation and analysis of the cytotoxic activity of eight germacranolides from this plant [27].

**Figure 1:**
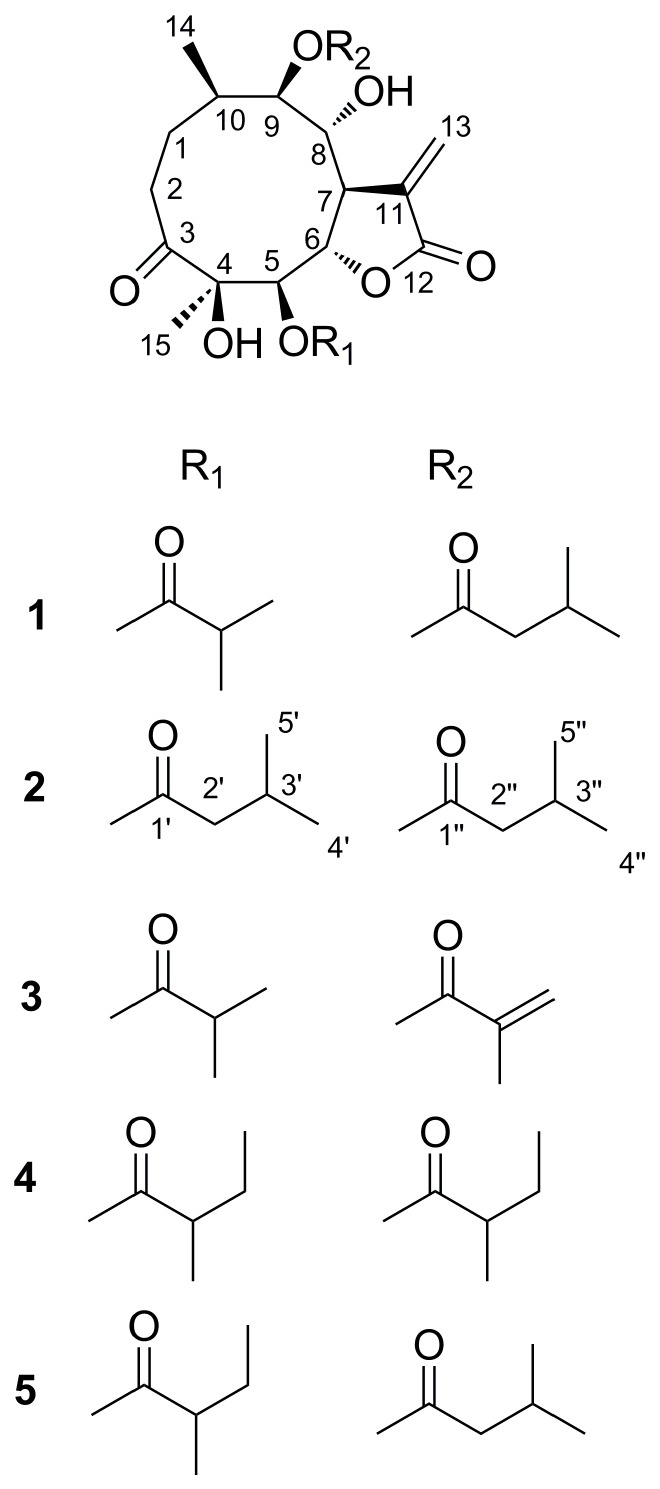
Chemical structures of compounds **1**–**5**.

As a part of our ongoing search for new bioactive products from medicinal plants in China, three new, and two known highly oxygenated germacranolides representing other subtype (Figure 1), were isolated from the whole plant of *C. divaricatum*. In this paper, a structural elucidation and bioactive evaluation of these compounds is presented.

## 2. Results and Discussion

### 2.1. Purification of Compounds **1**–**5**

The whole plant of *C. divaricatum* was extracted three times with EtOH–H_2_O (95:5). The five highly oxygenated germacranolides were isolated and purified via silica gel chromatograpy, Sephadex LH-20 gel chromatograpy, and semi-preparative High-Performance Liquid Chromatography (HPLC).

### 2.2. Structure Elucidation of Compounds **1**–**5**

**Figure 2:**
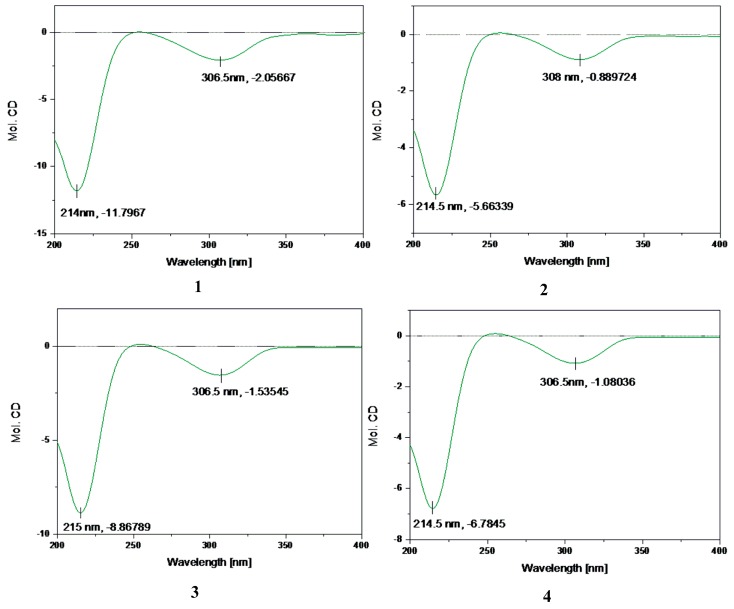
CD spectra of compounds **1**–**4**.

**Figure 3:**
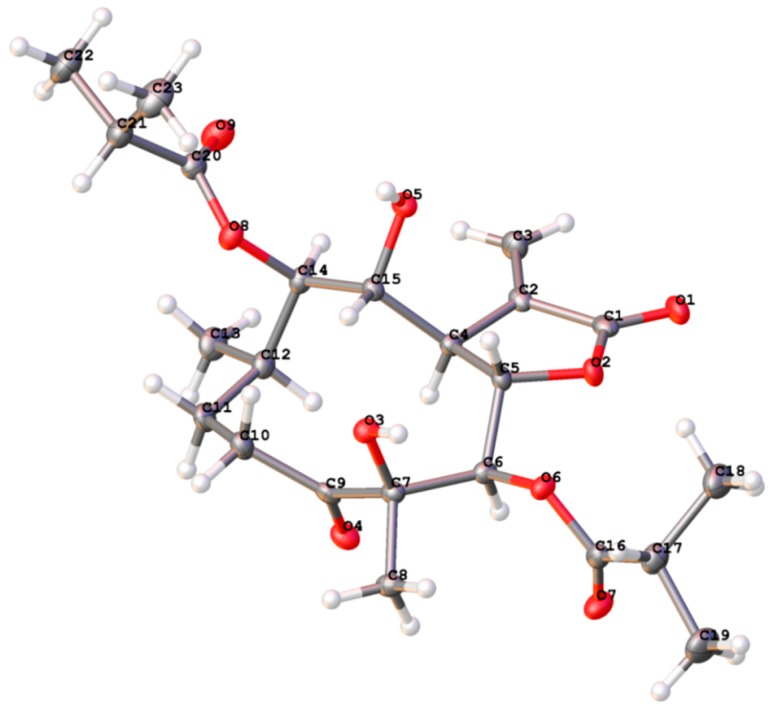
X-ray ORTEP drawing of **1**.

Compound **1** was identified as 4*β*,8*α*-dihydroxy-5*β*-isobutyryloxy-9*β*-3-methylbutyryloxy-3-oxo- germacran-6*α*, 12-olide (**1**), by comparison of its MS, NMR data, as well as optical rotation data, with reported data (supplementary materials Figure S1) [5]. However, its absolute configuration has not been determined. According to Beecham’s rule, the CD spectrum (Figure 2) of **1** exhibited a positive Cotton effect near 254 nm (*α*-methylene-*γ*-lactone region), supporting 6*S*, 7*R* configuration [21]. Fortunately, a suitable crystal was obtained for X-ray diffraction to confirm the absolute configuration. The X-ray crystallographic analysis [flack parameter: 0.08(17)] established unambiguously the absolute configuration of **1** to be 4*R*, 5*R*, 6*S*, 7*R*, 8*R*, 9*R* and 10*R* (Figure 3). Herein, the absolute configuration of **1** is reported for the first time.

**Figure 4:**
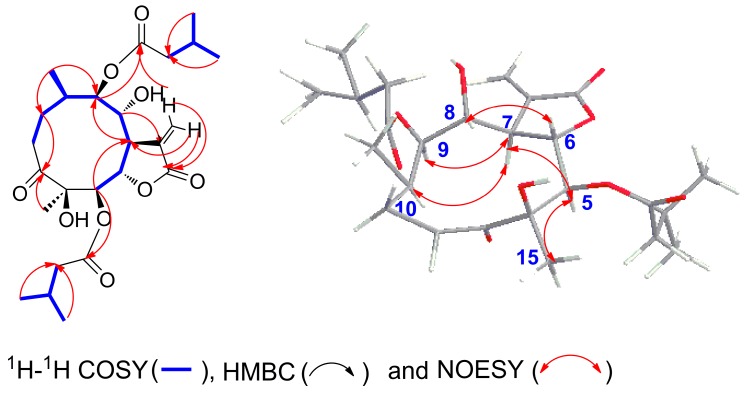
Key 2D correlations of compound **2**.

Compound **2** was obtained as white needles. The molecular formula was assigned as C_25_H_38_O_9_, on the basis of the positive-ion HRESIMS peak at *m*/*z* 505.2427 [M + Na]^+^, together with its ^1^H and ^13^C NMR data (Table 1). Its IR spectrum showed hydroxy (3458 cm^-1^) and carbonyl (1744 and 1718 cm^−1^) absorptions. The ^1^H and ^13^C NMR spectra of **2** showed an α-methylene-*γ*-lactone at *δ*_H_ 6.27 (1H, d, *J* = 3.0 Hz, Ha-13) and 5.62 (1H, d, *J* = 3.0 Hz, Hb-13), *δ*_C_ 132.6 (C-11), 123.9 (C-13) and 169.7 (C-12); three carbonyl carbons at *δ*_C_ 217.8 (C-3), 172.5 (C-1′) and 173.3 (C-1′′); one oxygenated tertiary carbon at 80.3 (C-4); four oxygenated methines at *δ*_H_ 5.36 (1H, dd, *J* = 8.5, 2.0 Hz, H-5), 4.60 (1H, dd, *J* = 8.5, 5.0 Hz, H-6), 4.35 (1H, d, *J* = 10.5 Hz, H-8), and 5.11 (1H, d, *J* = 10.5 Hz, H-9), *δ*_C_ 78.2 (C-5), 79.8 (C-6), 70.3 (C-8), and 78.7 (C-9); and two methyl groups at *δ*_H_ 0.92 (3H, d, *J* = 7.0 Hz, CH_3_-14), 1.18 (3H, s, CH_3_-15). These signals (^1^H and ^13^C NMR data) implied that the structure of **2** was similar to that of **1**, except that the isobutyryloxy group of **1** was replaced by a 3-methylbutyryloxy group at C-5 in **2**; this was further confirmed by the ^1^H-^1^H COSY, HSQC, and HMBC spectra (Figure 4). On the basis of these data, the planar structure of **2** was established.

The relative configuration of **2** was determined by analysis of ROESY data. The key NOE correlations of H-8/H-6, H-7/H-10, H-7/H-5, H-7/H-9, and H-5/H_3_-15 indicated that **2** had the same relative configuration as **1** (Figure 4). The CD spectrum of **2** showed positive Cotton effects at near 254 nm, which closely resembled those of **1**. Similar ROESY and CD data of **2** and **1** (Figure 2) assigned the absolute configuration of **2** as 4R, 5R, 6S, 7R, 8R, 9R, and 10R. Thus, the structure of compound **2**, named divarolide E, is defined as shown.

Compounds **3**–**4** possessed molecular formulas of C_23_H_32_O_9_ and C_25_H_38_O_9_, from their HRESIMS at *m*/*z* 475.1939 [M + Na]^+^ and *m*/*z* 505.2414 [M + Na]^+^ respectively. The ^1^H and ^13^C NMR data of **3**–**4** were similar to those of **1**, except that the 2-methacryloyloxy group at C-9 in **3** was observed in place of 3-methylbutyryloxy group in **1**, and an isobutyryloxy group at C-5 and the 3-methylbutyryloxy group at C-9 in **1** were replaced by two 2-methylbutyryloxy groups in **4**, respectively. These observations were confirmed by analyses of relevant ^1^H-^1^H COSY, HSQC and HMBC data (Table 1). The relative configurations of **3**–**4** were determined to be the same as those of **1**, by comparison of ROESY data for relevant protons. Similar CD data of **3**–**4** and **1** (Figure 2) revealed the same absolute configurations of **3**–**4** as that of **1**. Thus, the structures of compounds **3**–**4** were established as shown, and named divarolide F and divarolide G respectively.

The structure of the known compound (**5**) was identified as 4*β*,8*α*-dihydroxy-5*β*-2-methylbutyryloxy-9*β*-3-methylbutyryloxy-3-oxo-germacran-7*β*, 12*α*-olide [5], by comparison of its spectroscopic data with reported data.

### 2.3. In Vitro Cytotoxic Activities of Compounds **1**–**5**

Compounds **1**–**5** were evaluated for their cytotoxic activity against human cervical cancer (HeLa), hepatocellular cancer (Hep G2), and lung cancer (A549) cell lines (Table 2). The new compound **2,** and the known compound **5**, exhibited cytotoxicity against Hep G2 (IC_50_ values of 7.47 μM) and HeLa (IC_50_ values of 16.82 μM) cell lines, and the IC_50_ values were lower than those of the positive control *cis*-platin (IC_50_ values of 13.03, and 15.34 μM respectively). In addition, **1** and **2** also displayed strong cytotoxicity against Hep G2, with an IC_50_ value of 16.98 μM, and HeLa with an IC_50_ value of 16.82 μM.

## 3. Materials and Methods

### 3.1. General Experimental Procedures

Optical rotations were measured on a Perkin-Elmer 241 polarimeter (Perkin-Elmer, Waltham, MA, USA), and UV spectra were recorded on Shimadzu UV-2501 PC (Shimadzu, Kyoto, Japan). IR data were recorded using a Shimadzu FTIR-8400S spectrophotometer (Shimadzu, Kyoto, Japan). ^1^H and ^13^C NMR data were acquired using Bruker 500 and Bruker 600 instruments (Bruker, Rheinstetten, Germany), with solvent signals (CD_3_OD: *δ*_H_ 3.30/*δ*_C_ 49.0 ppm;) as references. HRESIMS data were acquired using a Q-TOF analyzer in SYNAPT HDMS system (Waters, Milford, MA, USA). X-ray diffraction data were collected on the Agilent GEMINI^TM^E instrument (CrysAlisPro software, Version 1.171.35.11; Agilent, Santa Clara, CA, USA). HPLC was performed using Waters 2535 system (Waters, Milford, MA, USA), with the following components: preparative column, a Daisogel-C_18_-100A (10 μm, 30 × 250 mm, ChuangXinTongHeng Sci. & Tech., Beijing, China) and a YMC-Pack ODS-A column (5 μm, 10 × 250 mm, YMC, Kyoto, Japan); and detector, Waters 2489 UV. Sephadex LH-20 (40–70 μm, Pharmacia Biotech AB, Uppsala, Sweden), silica gel (60–100, 100–200 and 200–300 mesh) and silica gel GF254 sheets (0.20–0.25 mm) (Qingdao Marine Chemical Plant, Qingdao, China) were used for column chromatography and TLC, respectively. TLC spots were visualized under UV light and by dipping into 5% H_2_SO_4_ in EtOH, followed by heating.

### 3.2. Plant Material

The whole plants of *C. divaricatum* were collected from EnShi, Hubei province (China) in August of 2013. They were identified by Prof. Ben-Gang Zhang of Institute of Medicinal Plant Development. A voucher specimen (No. 20130828) was deposited in the National Compound Library of Traditional Chinese Medicines, Institute of Medicinal Plant Development, Chinese Academy of Medical Sciences & Peking Union Medical College (CAMS & PUMC), Beijing, China.

### 3.3. Isolation and Purification of Compounds **1**–**5**

The air-dried plants (9 kg) were extracted three times (7 days each time) with EtOH–H_2_O (95:5) at room temperature. The combined extract was concentrated under reduced pressure to furnish a dark brown residue (570 g), which was suspended in H_2_O and partitioned in turn with petroleum ether (bp 60–90 °C), EtOAc, and n-BuOH. The EtOAc extract (207 g) was separated chromatographically on silica gel column (60–100 mesh, 16 × 20 cm) with a gradient mixture of CH_2_Cl_2_–MeOH (100:1, 60:1, 30:1, 15:1 and 6:1) as eluent. Five fractions were collected according to TLC analysis. Fraction A (CH_2_Cl_2_–MeOH, 100:1, 140 g) was separated by silica gel column chromatography (CC) (100–200 mesh, 16 × 20 cm) with petroleum ether–aceton (50:1, 25:1, 20:1, 15:1, 12:1, 10:1, 7:1, 5:1, 3:1 and 1:1) as eluent to give fractions A_1_–A_11_. Fraction A_10_ (petroleum ether–aceton, 3:1, 40 g) was separated by Sephadex LH-20 CC (5 × 200 cm, MeOH) to give Fr.A_10_S_1_–Fr.A_10_S_3_. Fraction A_10_S_2_ (20 g) was then subjected to MCI gel CC (6 × 50 cm) with a gradient mixture of MeOH–H_2_O (60:40, 80:20, and 100:0, 4000 mL each) to give three fractions (Fr.A_10_S_2_M_1_–Fr.A_10_S_2_M_3_).

Fraction A_10_S_2_M_2_ (13 g) was further separated chromatographically on silica gel column (200–300 mesh, 5 × 50 cm) with a gradient mixture of CH_2_Cl_2_–MeOH (150:1, 100:1, 50:1 and 20:1) as eluent, and a total of 86 fractions (Fr.A_10_S_2_M_2_-1–86, 200 mL each) were collected. Fraction A_10_S_2_M_2_-34–50 (1.5 g) were separated by preparative HPLC (20 mL/min, 70% MeOH in H_2_O) and semipreparative HPLC (2 mL/min, 52–75% MeOH in H_2_O for 25 min and followed by 75–95% MeOH in H_2_O for 10 min; 2 mL/min, 40–80% MeCN in H_2_O for 40 min) to yield **3** (5 mg). Fraction A_10_S_2_M_2_-74–79 (140 mg) were purified using semipreparative HPLC (2 mL/min, 60–80% MeOH in H_2_O for 25 min and followed by 80–90% MeOH in H_2_O for 20 min; 2 mL/min, 30–70% MeCN in H_2_O for 40 min) and to yield **1** (30 mg).

Fraction A_9_ (petroleum ether-Aceton, 5:1, 30 g) was separated by Sephadex LH-20 CC (5 × 200 cm, MeOH) to give Fr.A_9_S_1_–Fr.A_9_S_3_. Fraction A_9_S_2_ (20 g) was then subjected to MCI gel CC (6 × 50 cm) with a gradient mixture of MeOH–H_2_O (60:40, 80:20, and 100:0, 4000 mL each) to give three fractions (Fr.A_9_S_2_M_1_–Fr.A_9_S_2_M_3_). Fraction A_9_S_2_M_2_ (10 g) was further separated chromatographically on a silica gel column (100–200 mesh, 5 × 50 cm), with a gradient mixture of petroleum ether–Aceton (10:1, 7:1, 5:1, 3.5:1, 2:1 and 1:1) as eluent; a total of 200 fractions (Fr.A_9_S_2_M_2_-1–200, 50 mL each) were collected. Fraction A_9_S_2_M_2_-107–112 (2.5 g) were separated by silica gel column chromatography (CC) (200–300 mesh, 5 × 40 cm) with CH_2_Cl_2_–MeOH (150:1, 75:1, 30:1, and 15:1) as eluent to give Fr. A_9_S_2_M_2_-107–112-A_1_–Fr. A_9_S_2_M_2_-107–112-A_8_. Fraction A_9_S_2_M_2_-107–112–A_3_ (CH_2_Cl_2_–MeOH, 75:1, 500 mg) was further purified using semipreparative HPLC (2 mL/min, 65–90% MeOH in H_2_O for 40 min; 2 mL/min, 40–80% MeCN in H_2_O for 40 min) to yield **2** (4.5 mg), **4** (5 mg) and **5** (10 mg).

### 3.4. Characterization of Compounds **2–4**

Divarolide E (**2**): white needles (CH_3_OH), [α]${}_{D}^{20}$ –95.2 (c 0.125, MeOH); UV (MeOH) λmax (logε): 210 (3.38) nm, IR (KBr) ν_max_: 3458, 1744, 1718, 1661 cm^−1^; CD (MeOH) 215 (Δε −0.083), 308 (Δε −0.013) nm; HRESIMS (pos.): *m*/*z* 505.2427 [M + Na]^+^ (calcd for C_25_H_38_O_9_Na, 505.2414); ^1^H NMR and ^13^C NMR data, see Table 1.

Divarolide F (**3**): white needles (CH_3_OH), [α]${}_{D}^{20}$ –78.7 (c 0.150, MeOH); UV (MeOH) λmax (logε): 200 (4.68) nm, IR (neat) ν_max_: 3463, 1762, 1707, 1647 cm^−1^; CD (MeOH) 215 (Δε −0.122), 307 (Δε −0.021) nm; HRESIMS (pos.): *m*/*z* 475.1939 [M + Na]^+^ (calcd for C_23_H_32_O_9_Na, 475.1944); ^1^H NMR and ^13^C NMR data, see Table 1.

Divarolide G (**4**): white needles (CH_3_OH), [α]${}_{D}^{20}$ –84.7 (c 0.085, MeOH); UV (MeOH) λmax (logε): 209 (4.00) nm, IR (neat) ν_max_: 3440, 2969, 1740, 1660 cm^−1^; CD (MeOH) 215 (Δε −0.099), 307 (Δε −0.016) nm; HRESIMS (pos.): *m*/*z* 505.2414 [M + Na]^+^ (calcd for C_25_H_38_O_9_Na, 505.2414); ^1^H NMR and ^13^C NMR data, see Table 1.

### 3.5. X-ray Crystal Structure Analysis of Compound **1**

X-ray diffraction data were collected on the Agilent GEMINI^TM^E instrument (CrysAlisPro software, Version 1.171.35.11), with enhanced Cu Kα radiation (λ = 1.54184 Å). The structure was solved by direct methods and refined by full-matrix least-squares techniques (SHELXL-97). All non-hydrogen atoms were refined with anisotropic thermal parameters. Hydrogen atoms were located by geometrical calculations and from positions in the electron density maps. Crystallographic data (excluding structure factors) for **1** in this paper has been deposited with the Cambridge Crystallographic Data Centre (deposition number CCDC 1570798). Copies of the data can be obtained, free of charge, on application to CCDC, 12 Union Road, Cambridge CB2 1EZ, UK (fax: +44-12-23336033 or e-mail: deposit@ccdc.cam.ac.uk).

A colorless monoclinic crystal (0.22 × 0.18 × 0.03 mm) of **1** was grown from MeOH-H_2_O (3:1). Crystal data: C_24_H_36_O_9_, *M* = 471.55, *T* = 106.8 K, triclinic, space group*P*2_1_, a = 14.2950(7) Å, b = 9.5219(4) Å, c = 18.7748(11) Å, *α* = 90.00°, *β* = 104.713°, *γ* = 90.00°, *V* = 2471.7(2) Å^3^, *Z* = 4, *ρ* = 1.267 mg/mm^3^, *μ*(Cu Kα) = 0.805 mm^−1^, measured reflections = 18092, unique reflections = 9353 (R_int_ = 0.0470), largest difference peak/hole = 0.363/−0.247 e Å^−3^, and flack parameter = 0.08(17). The final Rindexes [*I* > 2*σ* (*I*)] were R_1_ = 0.0535, and wR_2_ = 0.1288. The final Rindexes (all data) were R_1_ = 0.0658, and wR_2_ = 0.1390. The goodness of fit on F^2^ was 1.007.

### 3.6. Cytotoxicity Assays of Compounds **1**–**5**

Cell cultures: Human A549, HepG2, and HeLa cell lines from Cancer Institute and Hospital of Chinese Academy of Medical Sciences (Beijing, China), were cultured in Dulbecco’s modified Eagle’s medium (DMEM, Gibco, CA, USA) supplemented with 10% (*v*/*v*) fetal calf serum (Gibco, CA, USA), penicillin G (Macgene, China) 100 units mL^−1^ and streptomycin (Macgene, China) 100 μg mL^−1^, at 37 °C under 5% CO_2_.

Cell viability assay: The assay was run in triplicate. In a 96-well plate, each well was plated with 2 × 10^4^ cells. After cell attachment overnight, the medium was removed, and each well was treated with 100 μL of medium containing 0.1% DMSO or different concentrations of the test compounds and the positive control *cis*-platin. The plate was incubated at 37 °C for 4 days in a humidified, 5% CO_2_ atmosphere. Cytotoxicity was determined using a modified 3-(4,5-dimethylthiazol-2-yl)-2,5-diphenyltetrazolium bromide (MTT) colorimetric assay [28]. After the addition of a 10 μL MTT solution (5 mg/mL), cells were incubated at 37 °C for 4 h. After adding 150 μL DMSO, cells were shaken to mix thoroughly. The absorbance of each well was measured at 540 nm in a Multiscan photometer. The IC_50_ values were calculated by Origin software and listed in Table 2.

Statistical analysis: Values were expressed as mean ± SD. Statistical analyses were performed using the Student’s *t*-test. Differences were considered significant when associated with a probability of 5% or less (*p* ≤ 0.05).

## 4. Conclusions

In conclusion, three new compounds (**2**–**4**), as well as two known compounds (**1** and **5**), were isolated from the whole plant of *C. divaricatum*. Structurally, all compounds contained a 5-membered *γ*-lactone ring fused to a circular 10-membered carbocycle. We obtained a set of isomers (**2**/**4**/**5**) from the same plant. The isolation of these isomers is a huge challenge because they are highly oxygenated and have similar structures. The absolute configuration of compound **1** was unambiguously established by X-ray diffraction. The other compounds with the same skeleton were determined by comparison of NOESY and CD data with those of **1**. Compounds **1** and **5** showed significant cytotoxicity against two human tumor cell lines. These findings are an important addition to the present knowledge on the structurally diverse and biologically significant germacranolide family.

## Figures and Tables

**Table 1:** NMR spectral data of **2**–**4**.

No.	2 ^a^		3 ^b^		4 ^a^	
	*δ*_C_, Type	*δ*_H_ (*J* in Hz)	*δ*_c_, Type	*δ*_H_ (*J* in Hz)	*δ*_c_, Type	*δ*_H_ (*J* in Hz)
1	25.3 CH_2_	1.79 m, 1.65 m	25.3 CH_2_	1.83 m, 1.71 m	25.3 CH_2_	1.87 m, 1.75 m
2	32.8 CH_2_	3.76 br d (7.5), 2.22 m	32.9 CH_2_	3.81 br d (7.5), 2.16 m	33.2 CH_2_	3.87 m, 2.29 m
3	217.8 C		217.6 C		217.6 C	
4	80.3 C		80.4 C		80.3 C	
5	78.2 CH	5.36 dd (8.5, 2.0)	78.1 CH	5.37 dd (9.6, 1.2)	78.1 CH	5.39 br d (9.5)
6	79.8 CH	4.60 dd (8.5, 5.0)	79.9 CH	4.65 dd (9.6, 6.0)	79.9 CH	4.69 dd (9.5,6.5)
7	41.5 CH	2.97 m	41.6 CH	3.01 m	41.7 CH	3.02 m
8	70.3 CH	4.35 d (10.5)	70.3 CH	4.43 d (10.2)	70.5 CH	4.40 d (10.0)
9	78.7 CH	5.11 d (10.5)	79.3 CH	5.18 d (10.2)	78.4 CH	5.15 d (10.0)
10	29.8 CH	2.15 m	30.1 CH	2.21 m	30.0 CH	2.23 m
11	132.6 C		132.7 C		132.7 C	
12	169.6 C		169.6 C		169.5 C	
13	123.9 CH_2_	6.27 d (3.0), 5.62 d (3.0)	123.8 CH_2_	6.30 d (3.0) 5.67 d (3.0)	123.8 CH_2_	6.32 d (3.0) 5.67 d (3.0)
14	20.0 CH_3_	0.92 d (7.0)	19.9 CH_3_	0.94 d (6.6)	20.0 CH_3_	0.98 d (6.5)
15	23.4 CH_3_	1.18 s	23.3 CH_3_	1.21 s	23.5 CH_3_	1.24 s
1′	172.5 C		176.3 C		175.9 C	
2′	42.7 CH_2_	2.31 d (7.0), 2.26 o	33.9 CH	2.67 m	41.3 CH	2.52 m
3′	25.3 CH	2.09 o ^c^	18.0 CH_3_	1.21 d (6.6)	26.3 CH_2_	1.76 o, 1.52 o
4′	21.3 CH_3_	0.96 d (6.0)	17.9 CH_3_	1.20 d (6.6)	16.1 CH_3_	1.24 d (7.0)
5′	21.4 CH_3_	0.95 d (6.0)			10.7 CH_3_	0.98 t (7.0)
1′′	173.3 C		167.2 C		176.7 C	
2′′	43.0 CH_2_	2.26 o, 2.06 o	136.4 C		41.5 CH	2.52 m
3′′	25.4 CH	2.09 o	124.7 CH_2_	5.63 dq (3.6, 1.8), 6.13 dq (3.6, 1.8)	26.2 CH_2_	1.76 o, 1.52 o
4′′	21.4 CH_3_	0.96 d (6.5)	17.1 CH_3_	1.96 br s	16.2 CH_3_	1.26 d (7.0)
5′′	21.4 CH_3_	0.95 d (6.5)			10.6 CH_3_	0.96 t (7.0)

^a^ Measured at 500 MHz in methanol-*d*_4_; ^b^ Measured at 600 MHz in methanol-*d*_4_; ^c^ Overlapped with other signals.

**Table 2:** In Vitro Cytotoxic Activities of Compounds **1**–**5**.

Compounds	IC_50_ (μM)		
	A549	HepG2	Hela
**1**	>40	16.98 ± 2.23	29.39 ± 0.17
**2**	30.70 ± 0.51	7.47 ± 0.21	16.82 ± 0.27
**3**	>40	>40	>40
**4**	>40	>40	>40
**5**	>40	31.64 ± 0.16	11.63 ± 1.00
***cis*-platin**	7.90 ± 0.23	13.03 ± 1.49	15.34 ± 0.35

Values were mean ± SD; Cis-platin, positive control; Cell lines: A549: lung cancer, Hep G2: hepatocellular cancer, and HeLa: cervical cancer.

## Supplementary Materials

The Supplementary Materials are available online.
