# Supplementary material for: Three New Highly Oxygenated Germacranolides from Carpesium Divaricatum and Their Cytotoxic Activity
Source: Molecules. 2018 May 3;23(5):1078. doi: 10.3390/molecules23051078 (PMC6102670; doi:10.3390/molecules23051078)
Supplement: Supplementary file 1 [file molecules-23-01078-s001.zip › molecules-293081-SI.docx]

Supplementary Information

Three new highly oxygenated germacranolides from *Carpesium divaricatum* and their cytotoxic activity

Tao Zhang,^1^ Qiu-Bo Zhang,^1^ Jin-Guang Si,^1^ Jia-Huan Chen,^1, 2^ Gang Ding,^1^ Hong-Wu Zhang,^1^ Hong-Mei Jia,^1^ and Zhong-Mei Zou^1*^

^1^ Institute of Medicinal Plant Development, Chinese Academy of Medical Sciences and Peking Union Medical College, Beijing 100193, P. R. China.

^2^ School of Traditional Chinese Medicine, Shenyang Pharmaceutical University, Shenyang 110016, P. R. China

* Corresponding Author

Zhong-Mei Zou, Tel/Fax: 86-10-57833290,

*E-mail address*: [zmzou@implad.ac.cn](mailto:zmzou@implad.ac.cn).

**Contents of Supplementary Information**

|  | contents |
| --- | --- |
| Fig. S1.1 | ^1^H NMR spectrum (600 MHz) of compound **1** in CD_3_OD |
| Fig. S1.2 | ^13^C NMR spectrum (150 MHz) of compound **1** in CD_3_OD |
| Fig. S1.3 | ^1^H-^1^H COSY spectrum (600 MHz) of compound **1** in CD_3_OD |
| Fig. S1.4 | HSQC spectrum (600 MHz) of compound **1** in CD_3_OD |
| Fig. S1.5 | HMBC spectrum (600 MHz) of compound **1** in CD_3_OD |
| Fig. S1.6 | HRESIMS spectrum of compound **1** |
| Fig. S1.7 | CD spectrum of compound **1** |
| Fig. S1.8 | X-ray ORTEP drawing of **1** |
| Fig. S2.1 | ^1^H NMR spectrum (600 MHz) of divarolide E (**2**) in CD_3_OD |
| Fig. S2.2 | ^13^C NMR spectrum (150 MHz) of divarolide E (**2**) in CD_3_OD |
| Fig. S2.3 | ^1^H-^1^H COSY spectrum (600 MHz) of divarolide E (**2**) in CD_3_OD |
| Fig. S2.4 | HSQC spectrum (600 MHz) of divarolide E (**2**) in CD_3_OD |
| Fig. S2.5 | HMBC spectrum (600 MHz) of divarolide E (**2**) in CD_3_OD |
| Fig. S2.6 | NOESY spectrum (600 MHz) of divarolide E (**2**) in CD_3_OD |
| Fig. S2.7 | UV spectrum of divarolide E (**2**) |
| Fig. S2.8 | IR spectrum of divarolide E (**2**) |
| Fig. S2.9 | HRESIMS spectrum of divarolide E (**2**) |
| Fig. S2.10 | CD spectrum of divarolide E (**2**) |
| Fig. S3.1 | ^1^H NMR spectrum (600 MHz) of divarolide F (**3**) in CD_3_OD |
| Fig. S3.2 | ^13^C NMR spectrum (150 MHz) of divarolide F (**3**) in CD_3_OD |
| Fig. S3.3 | ^1^H-^1^H COSY spectrum (600 MHz) of divarolide F (**3**) in CD_3_OD |
| Fig. S3.4 | HSQC spectrum (600 MHz) of divarolide F (**3**) in CD_3_OD |
| Fig. S3.5 | HMBC spectrum (600 MHz) of divarolide F (**3**) in CD_3_OD |
| Fig. S3.6 | ROESY spectrum (600 MHz) of divarolide F (**3**) in CD_3_OD |
| Fig. S3.7 | UV spectrum of divarolide F (**3**) |
| Fig. S3.8 | IR spectrum of divarolide F (**3**) |
| Fig. S3.9 | HRESIMS spectrum of divarolide F (**3**) |
| Fig. S3.10 | CD spectrum of divarolide F (**3**) |
| Fig. S4.1 | ^1^H NMR spectrum (500 MHz) of divarolide G (**4**) in CD_3_OD |
| Fig. S4.2 | ^13^C NMR spectrum (125 MHz) of divarolide G (**4**) in CD_3_OD |
| Fig. S4.3 | ^1^H-^1^H COSY spectrum (500 MHz) of divarolide G (**4**) in CD_3_OD |
| Fig. S4.4 | HSQC spectrum (500 MHz) of divarolide G (**4**) in CD_3_OD |
| Fig. S4.5 | HMBC spectrum (500 MHz) of divarolide G (**4**) in CD_3_OD |
| Fig. S4.6 | NOESY spectrum (600 MHz) of divarolide G (**4**) in CD_3_OD |
| Fig. S4.7 | UV spectrum of divarolide G (**4**) |
| Fig. S4.8 | IR spectrum of divarolide G (**4**) |
| Fig. S4.9 | HRESIMS spectrum of divarolide G (**4**) |
| Fig. S4.10 | CD spectrum of divarolide G (**4**) |


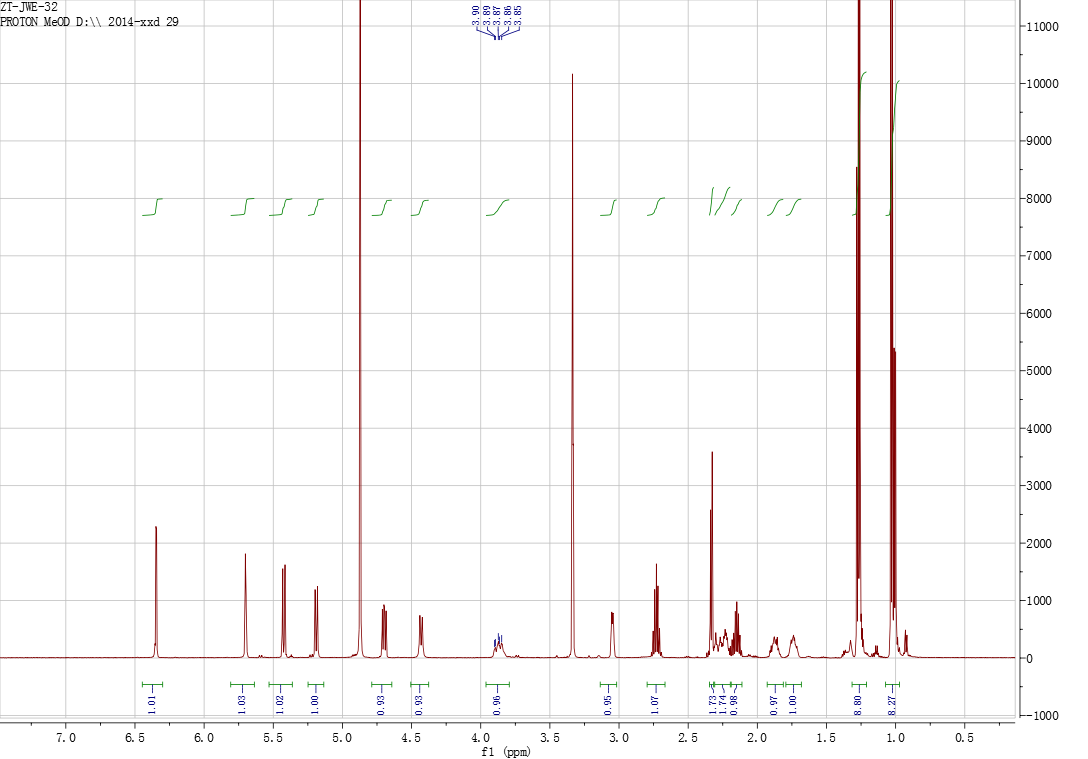


Fig. S1.1 ^1^H NMR spectrum (600 MHz) of compound **1** in CD_3_OD


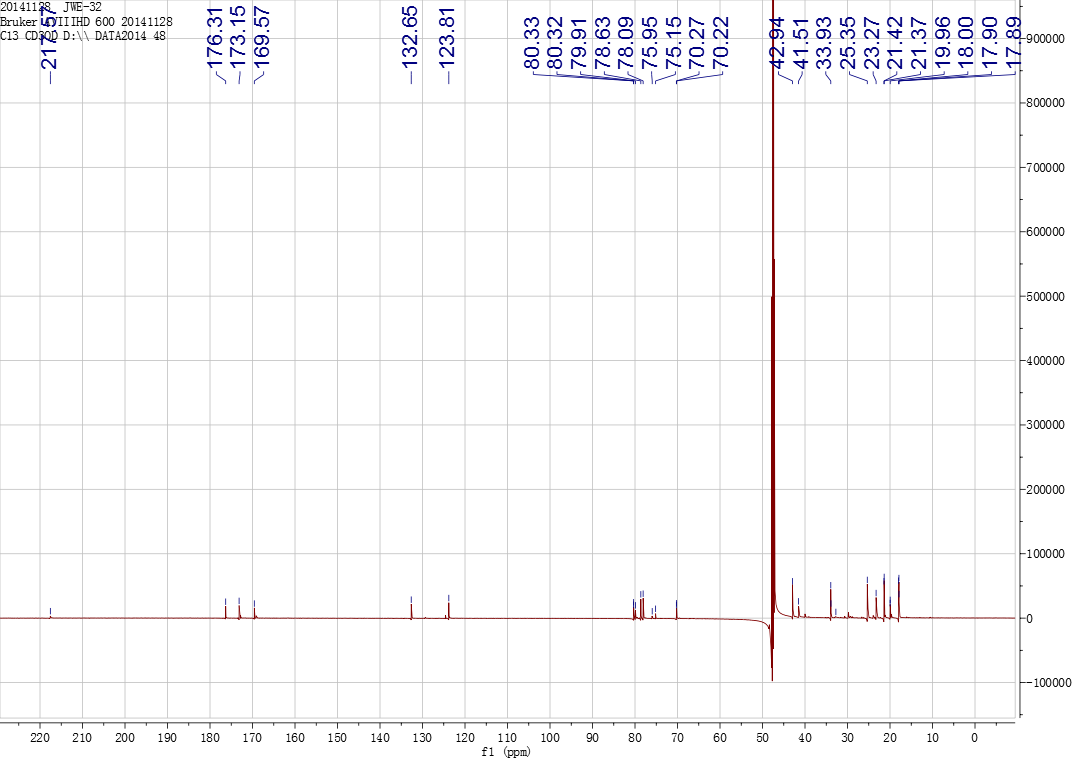


Fig. S1.2 ^13^C NMR spectrum (150 MHz) of compound **1** in CD_3_OD


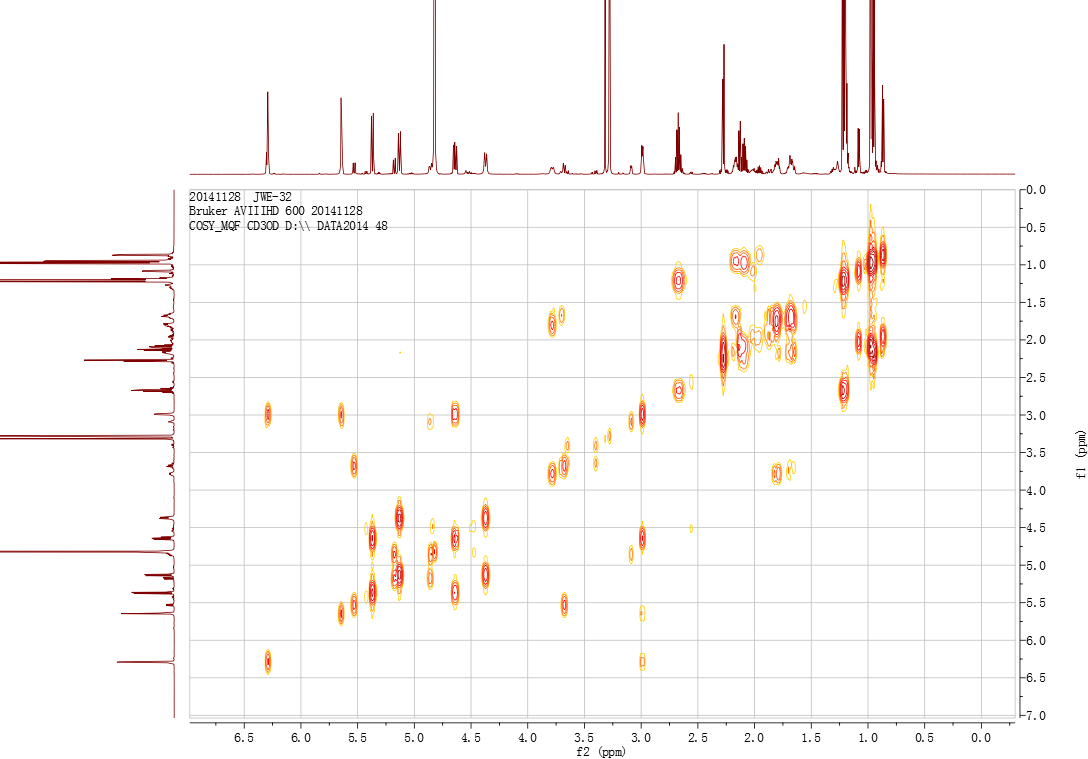


Fig. S1.3 ^1^H-^1^H COSY spectrum (600 MHz) of compound **1** in CD_3_OD


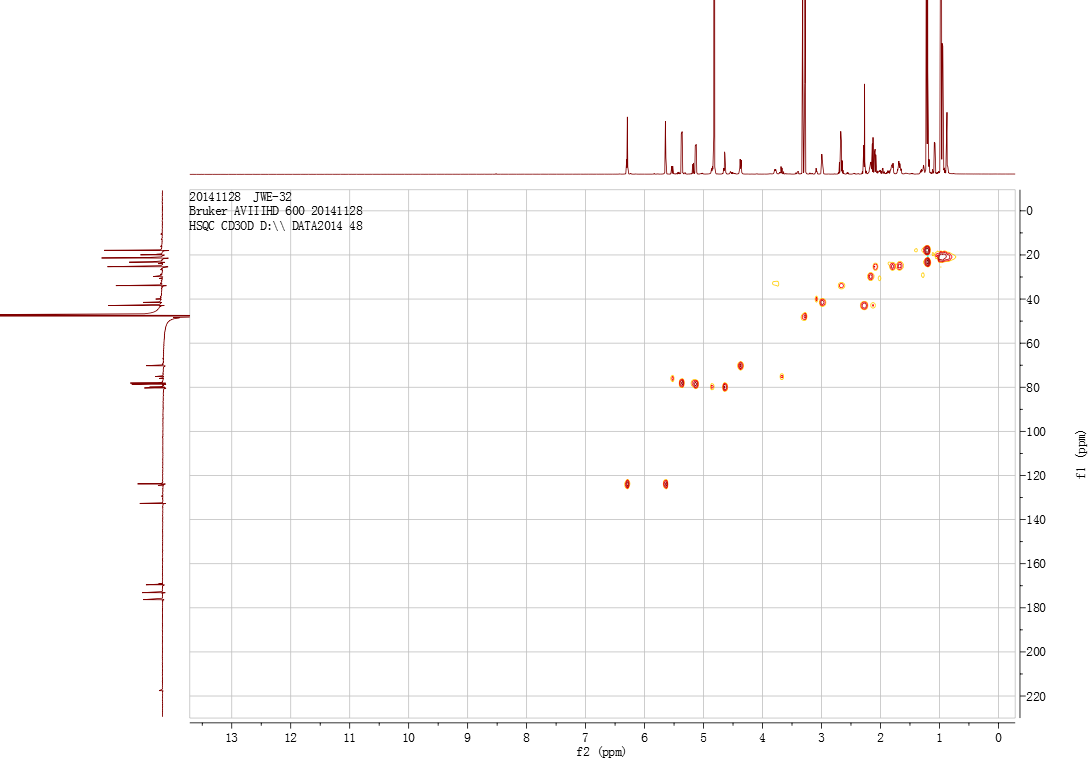


Fig. S1.4 HSQC spectrum (600 MHz) of compound **1** in CD_3_OD


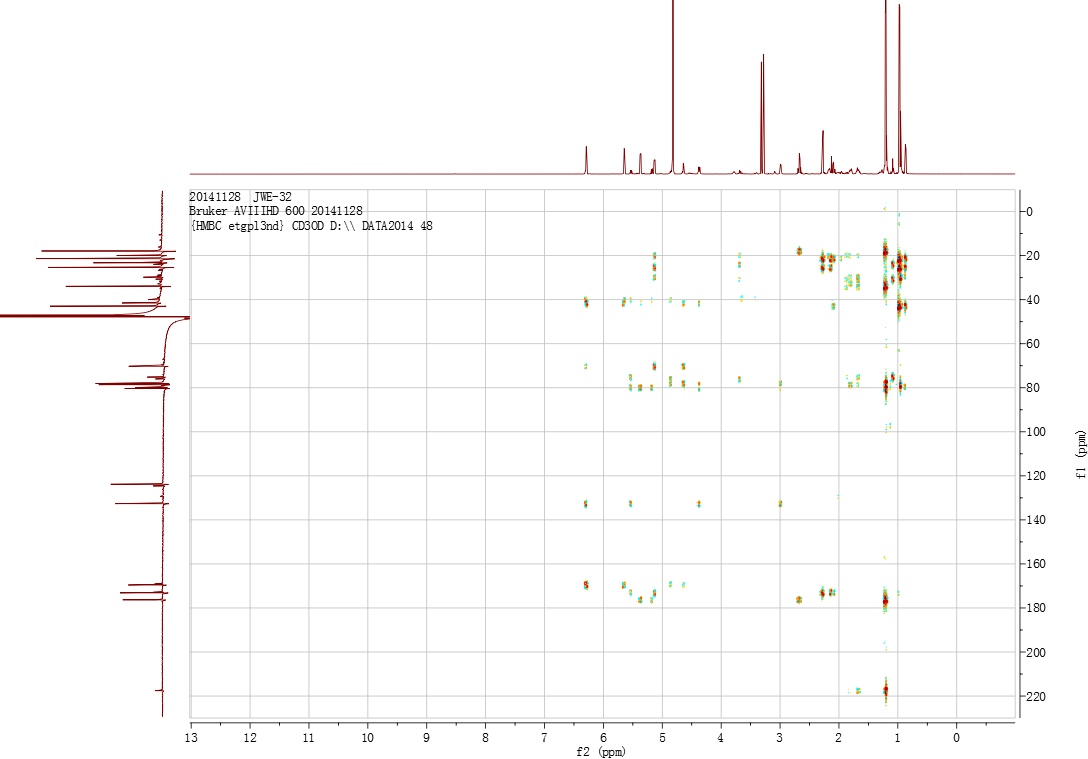


Fig. S1.5 HMBC spectrum (600 MHz) of compound **1** in CD_3_OD

Fig. S1.6 HRESIMS spectrum of compound **1**

Fig. S1.7 CD spectrum of compound **1**


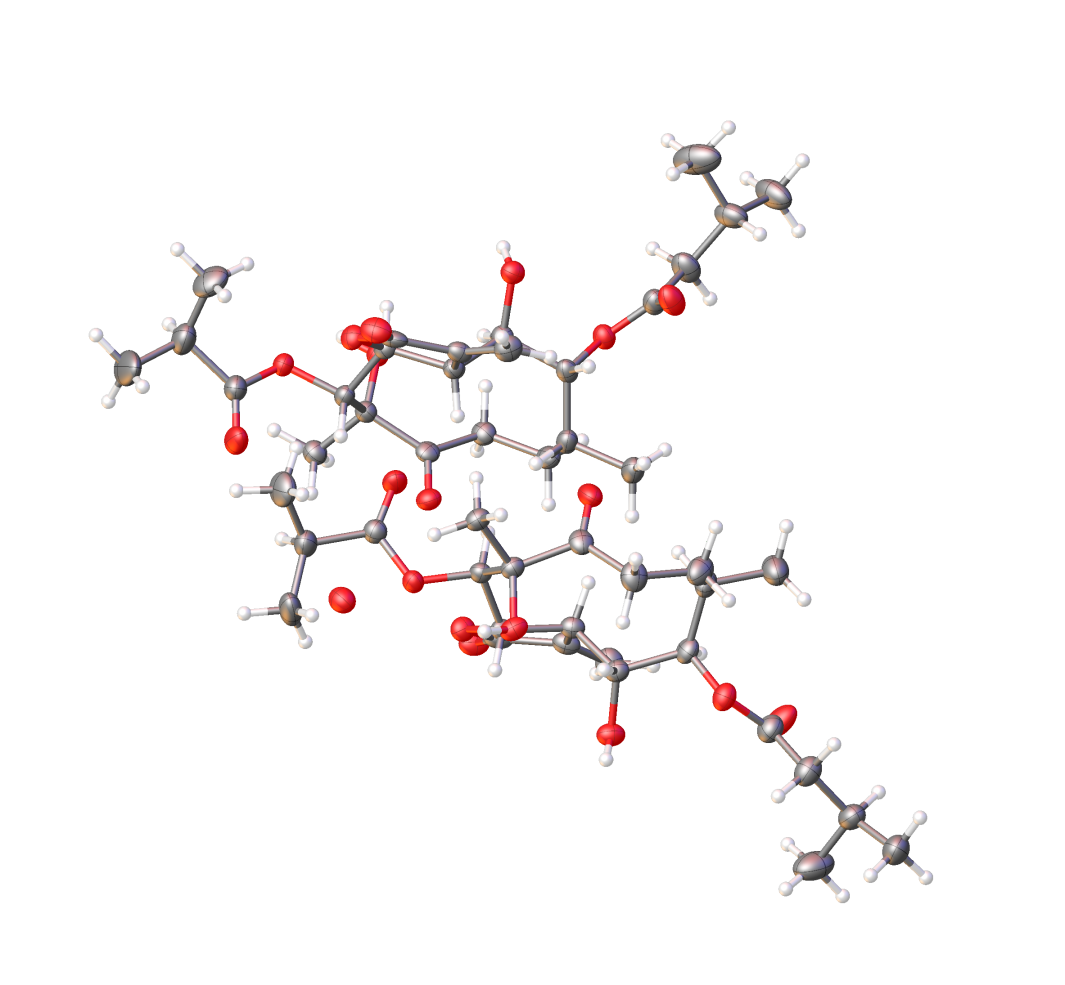


Fig. S1.8 X-ray ORTEP drawing of **1**


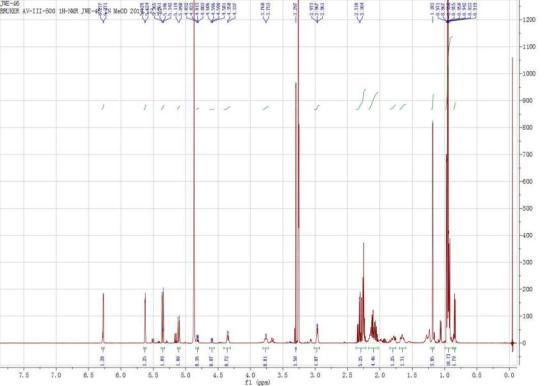


Fig. S2.1 ^1^H NMR spectrum (600 MHz) of divarolide E (**2**) in CD_3_OD


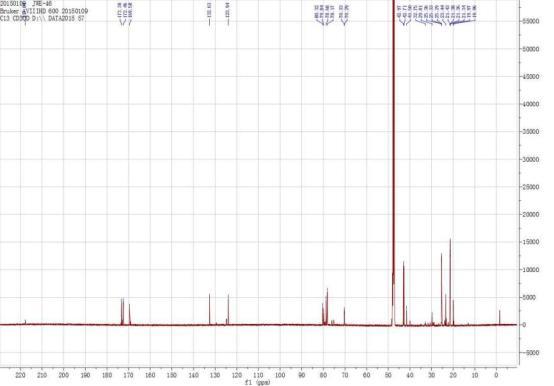


Fig. S2.2 ^13^C NMR spectrum (150 MHz) of divarolide E (**2**) in CD_3_OD


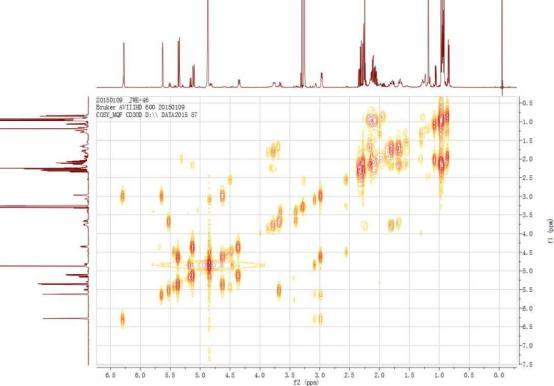


Fig. S2.3 ^1^H-^1^H COSY spectrum (600 MHz) of divarolide E (**2**) in CD_3_OD


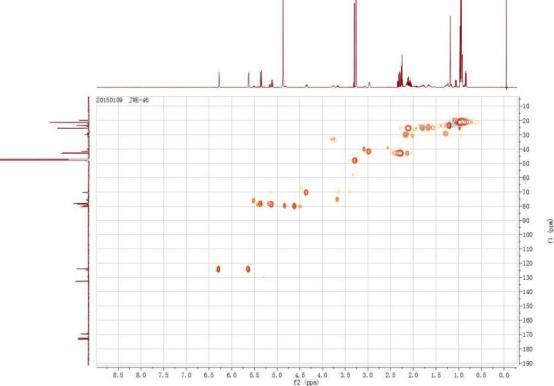


Fig. S2.4 HSQC spectrum (600 MHz) of divarolide E (**2**) in CD_3_OD


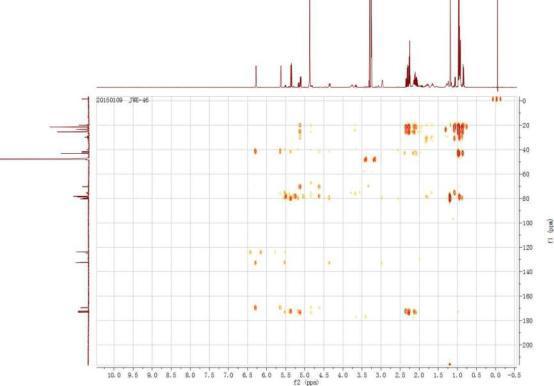
 Fig. S2.5 HMBC spectrum (600 MHz) of divarolide E (**2**) in CD_3_OD


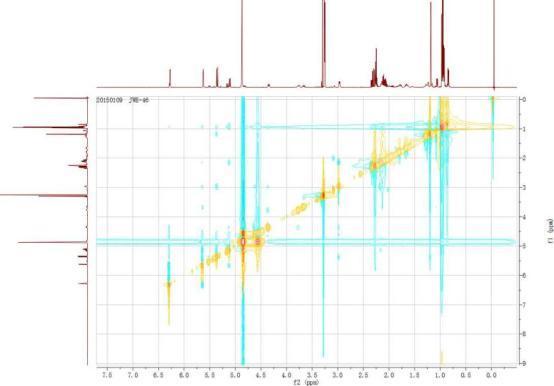


Fig. S2.6 NOESY spectrum (600 MHz) of divarolide E (**2**) in CD_3_OD


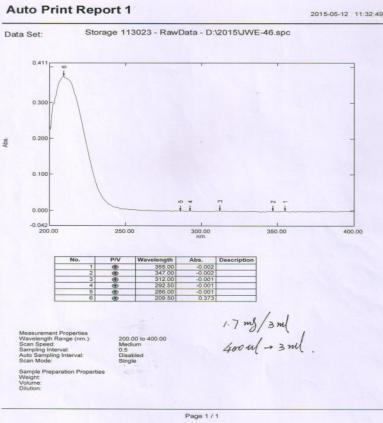


Fig. S2.7 UV spectrum of divarolide E (**2**)


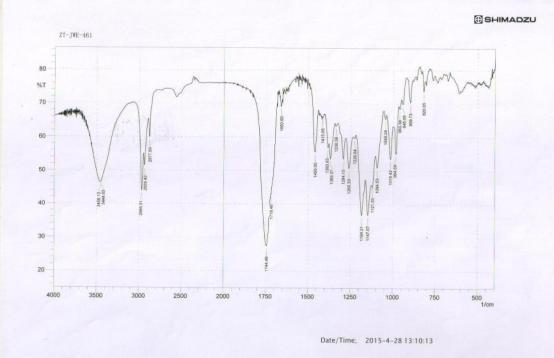


Fig. S2.8 IR spectrum of divarolide E (**2**)

Fig. S2.9 HRESIMS spectrum of divarolide E (**2**)

Fig. S2.10 CD spectrum of divarolide E (**2**)


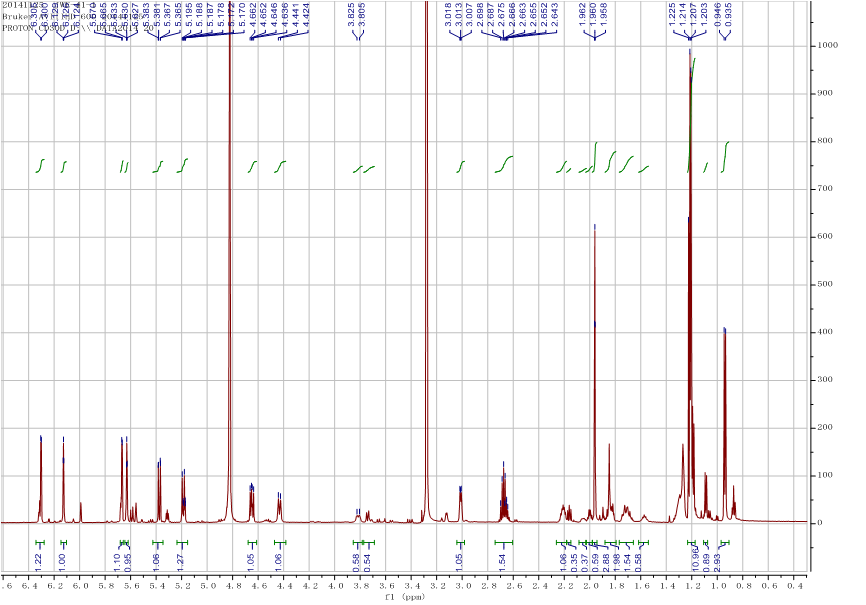


Fig. S3.1 ^1^H NMR spectrum (600 MHz) of divarolide F (**3**) in CD_3_OD


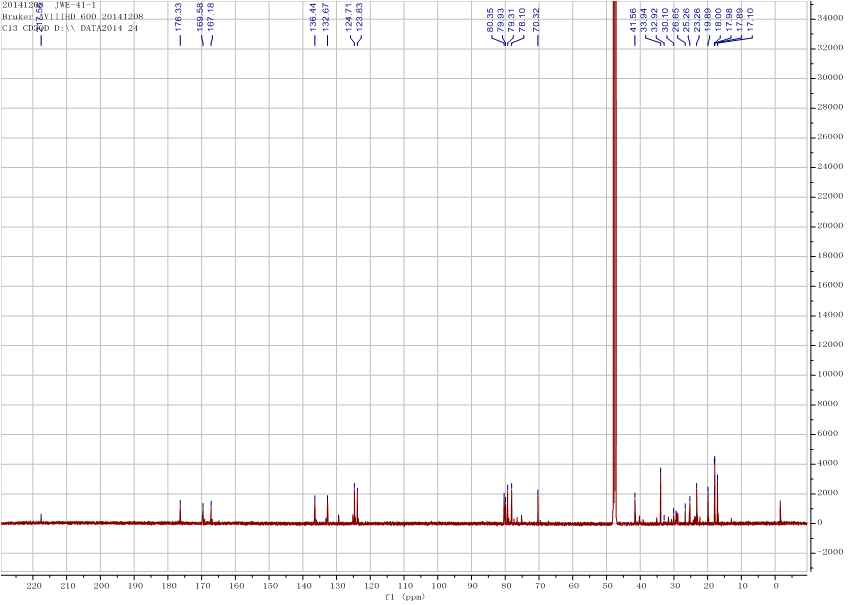


Fig. S3.2 ^13^C NMR spectrum (150 MHz) of divarolide F (**3**) in CD_3_OD


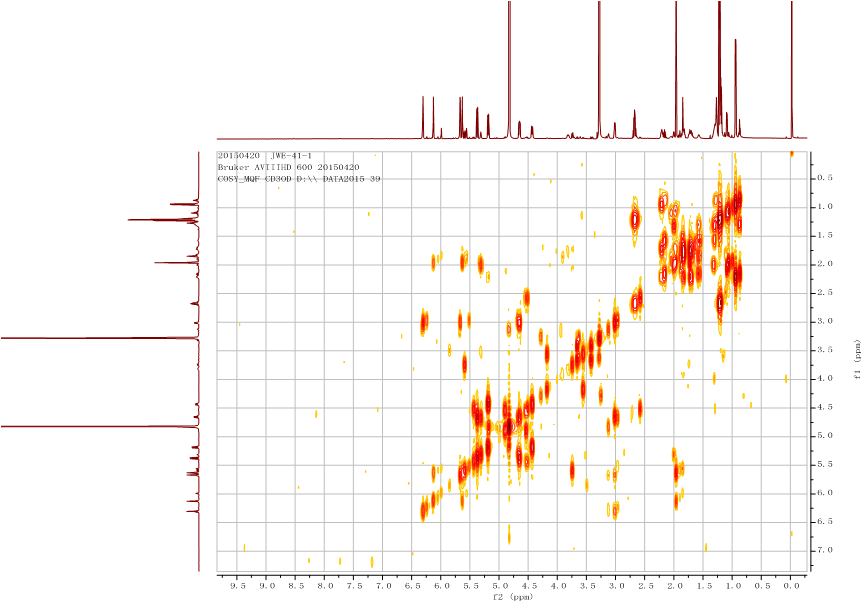


Fig. S3.3 ^1^H-^1^H COSY spectrum (600 MHz) of divarolide F (**3**) in CD_3_OD


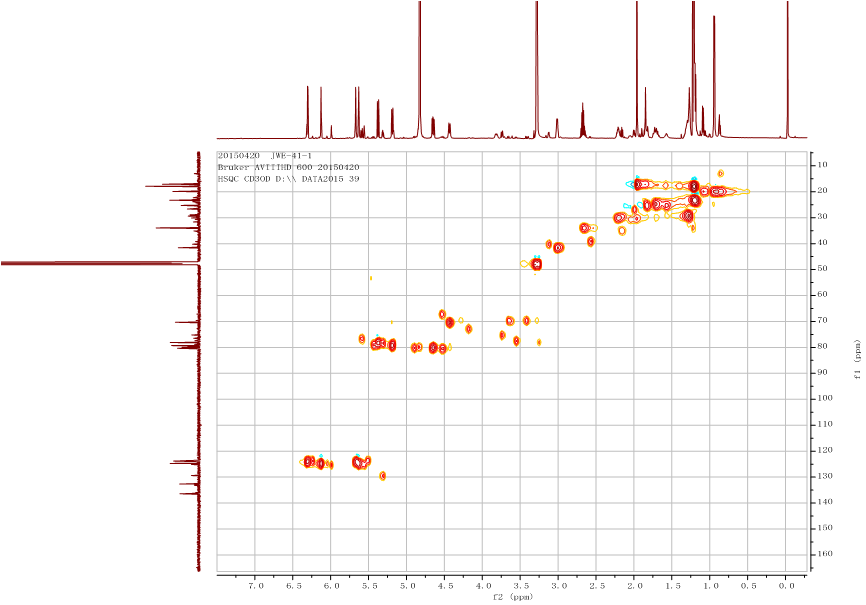


Fig. S3.4 HSQC spectrum (600 MHz) of divarolide F (**3**) in CD_3_OD


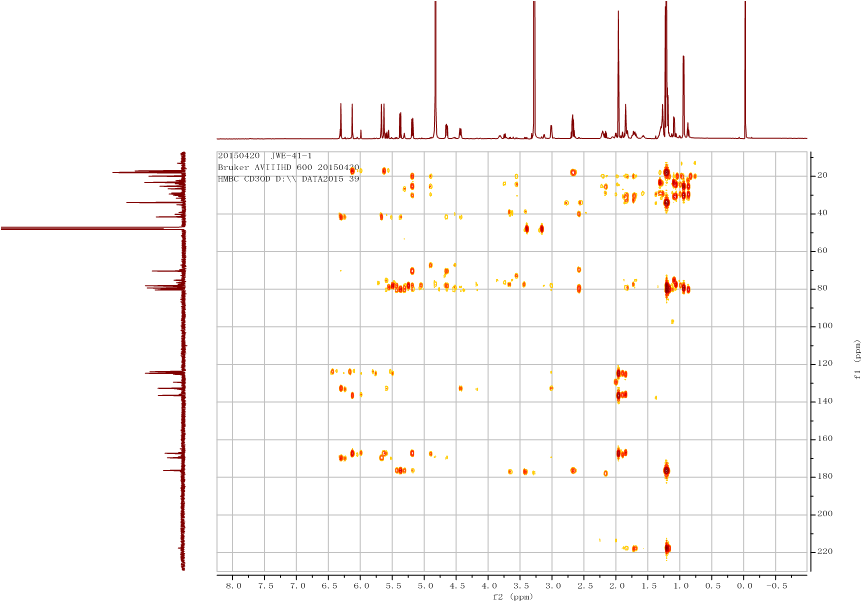


Fig. S3.5 HMBC spectrum (600 MHz) of divarolide F (**3**) in CD_3_OD


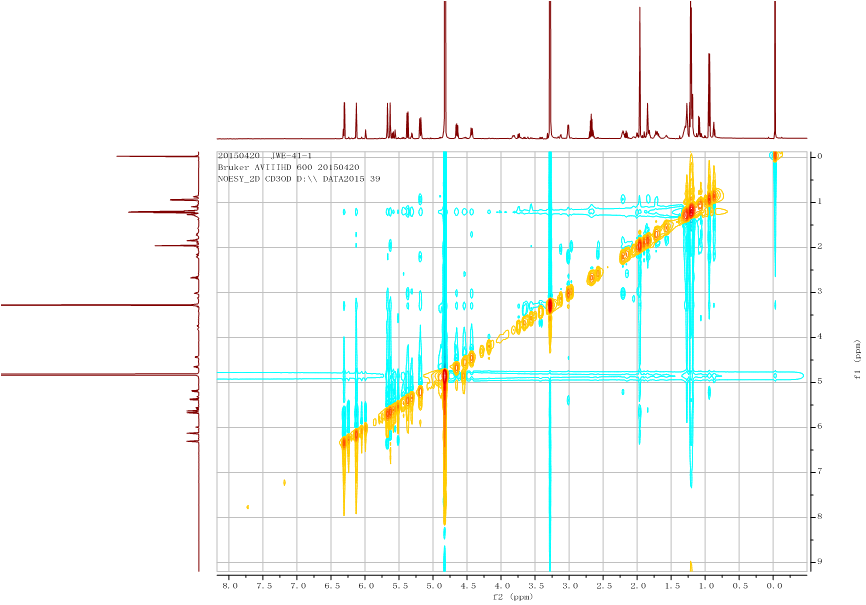


Fig. S3.6 NOESY spectrum (600 MHz) of divarolide F (**3**) in CD_3_OD


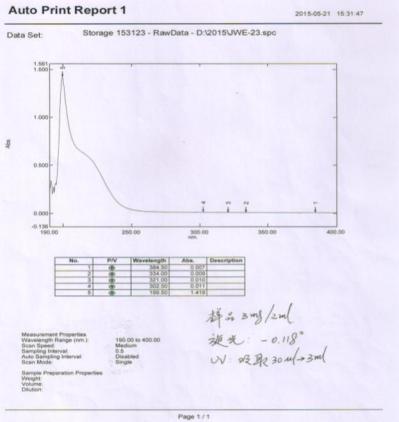


Fig. S3.7 UV spectrum of divarolide F (**3**)


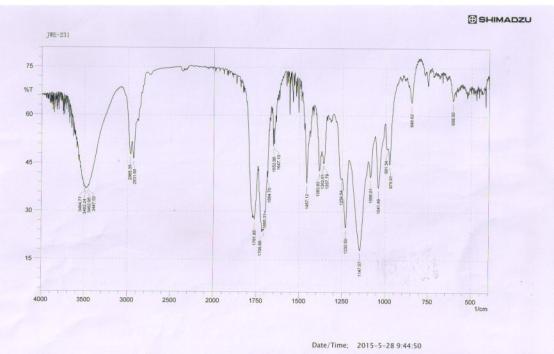


Fig. S3.8 IR spectrum of divarolide F (**3**)

Fig. S3.9 HRESIMS spectrum of divarolide F (**3**)

Fig. S3.10 CD spectrum of divarolide F (**3**)


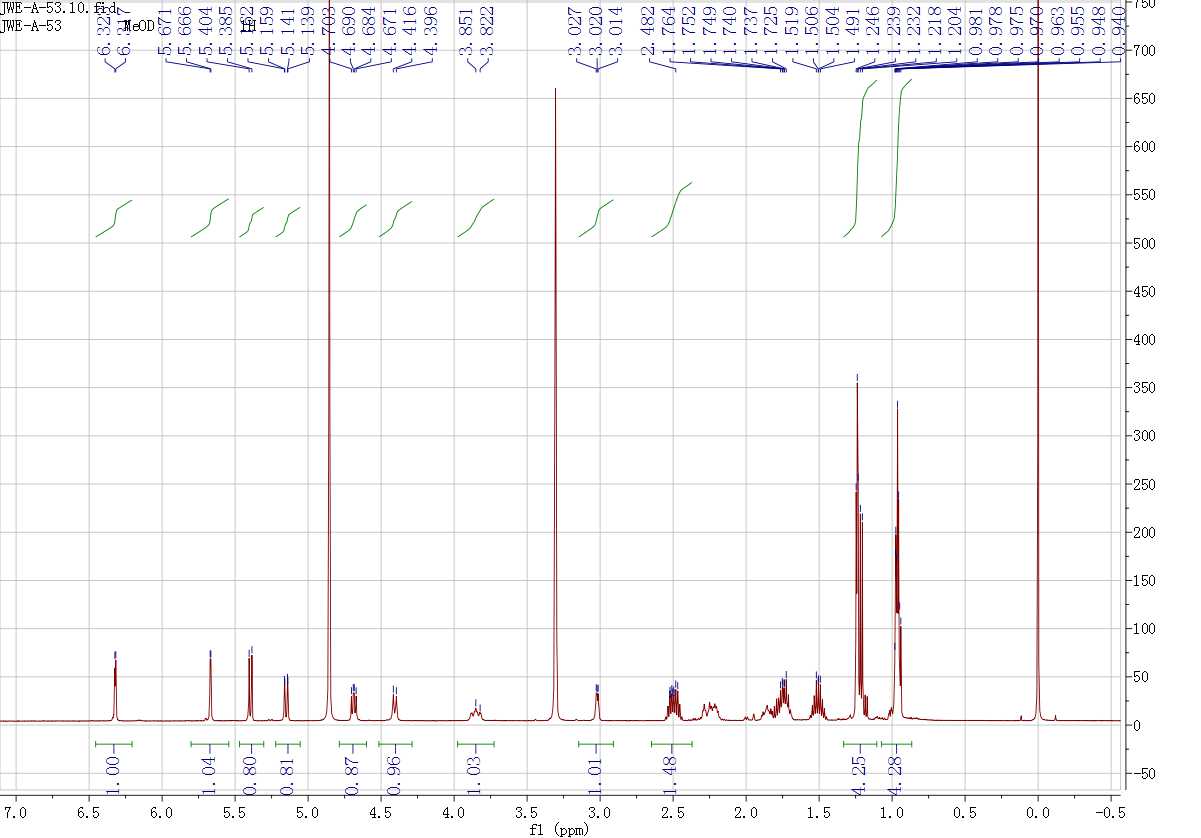


Fig. S4.1 ^1^H NMR spectrum (500 MHz) of divarolide G (**4**) in CD_3_OD


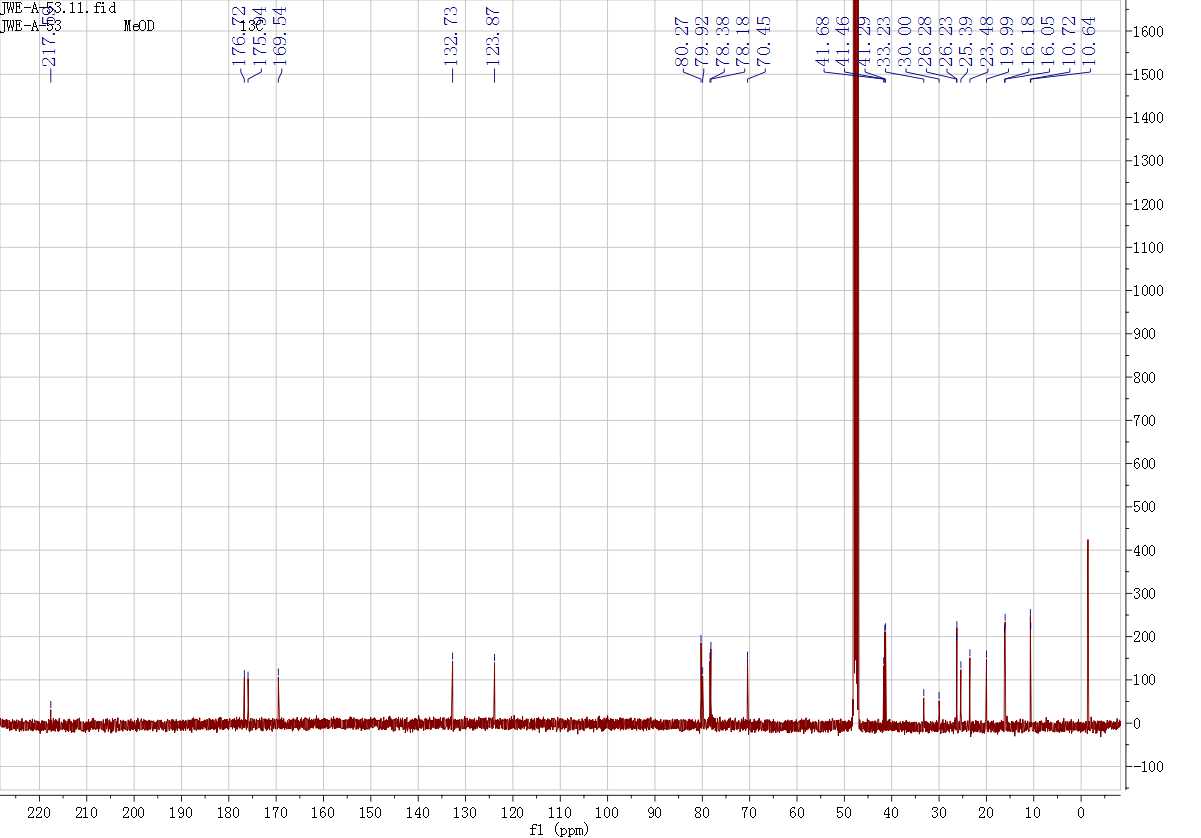


Fig. S4.2 ^13^C NMR spectrum (125 MHz) of divarolide G (**4**) in CD_3_OD


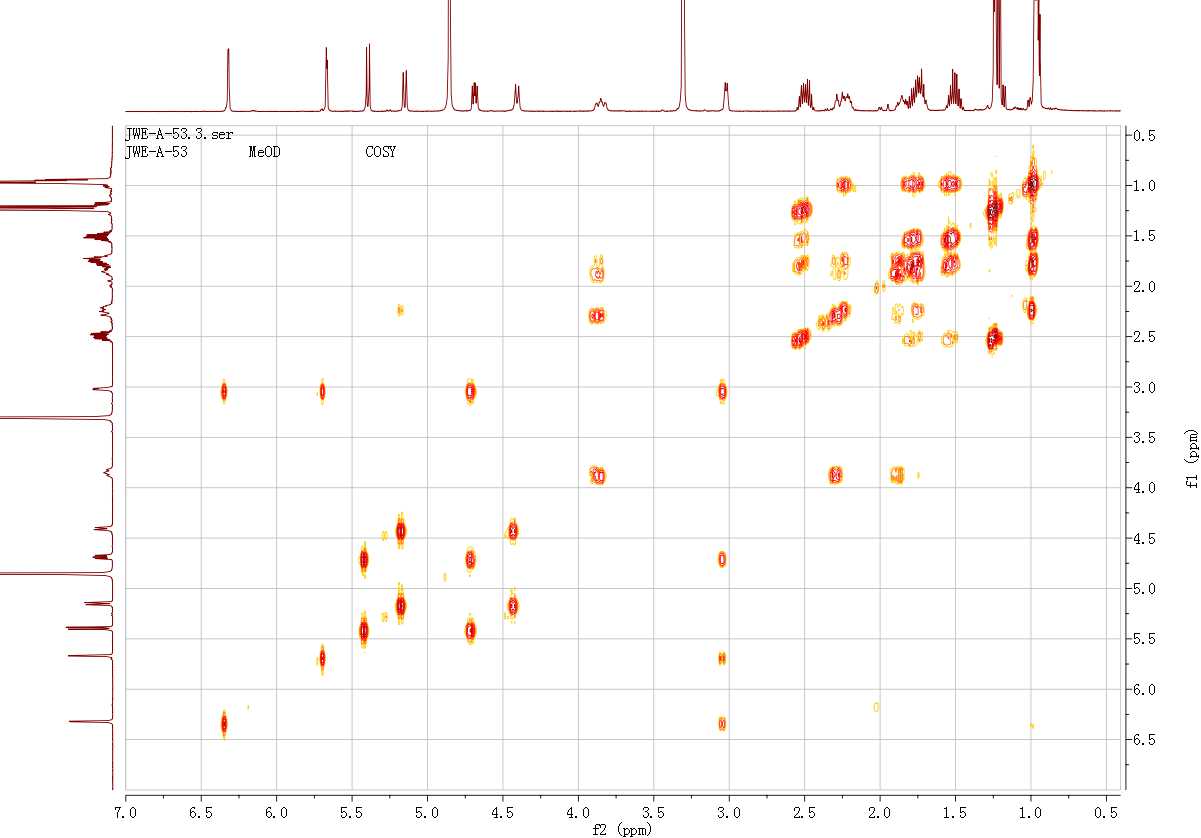


Fig. S4.3 ^1^H-^1^H COSY spectrum (500 MHz) of divarolide G (**4**) in CD_3_OD


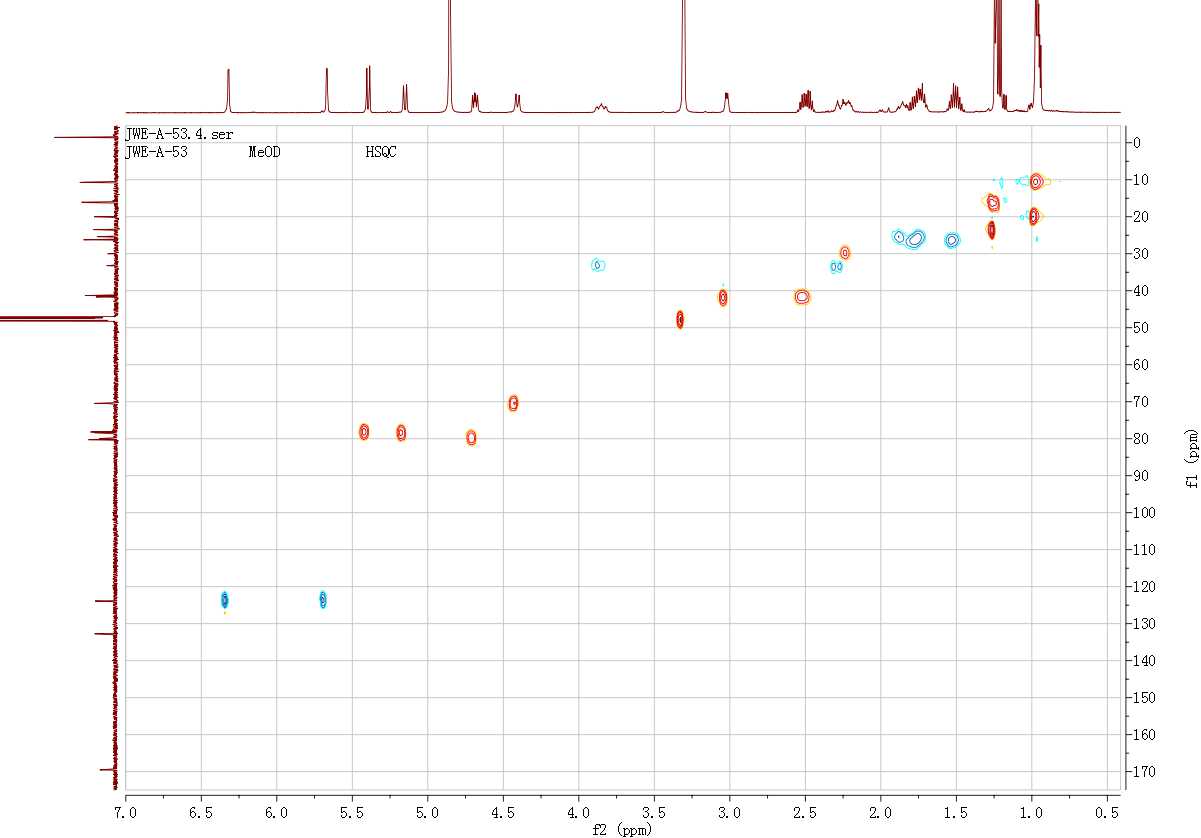


Fig. S4.4 HSQC spectrum (500 MHz) of divarolide G (**4**) in CD_3_OD


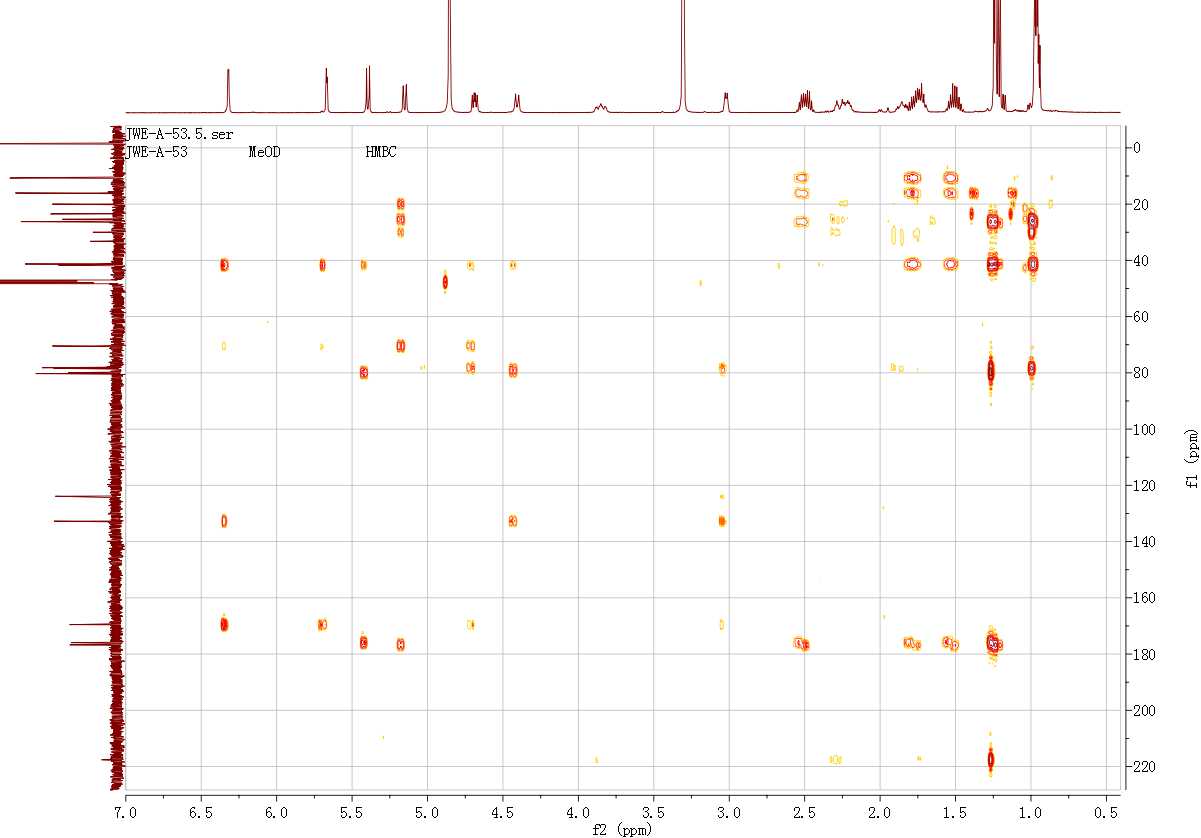


Fig. S4.5 HMBC spectrum (500 MHz) of divarolide G (**4**) in CD_3_OD


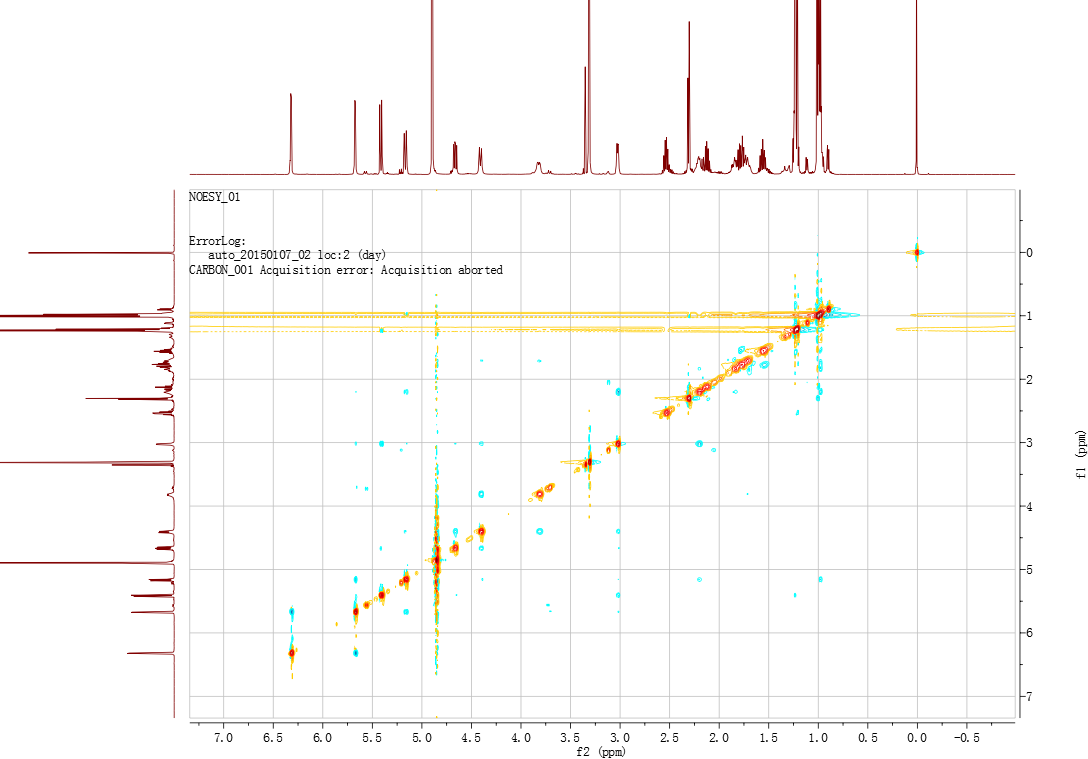


Fig. S4.6 NOESY spectrum (600 MHz) of divarolide G (**4**) in CD_3_OD


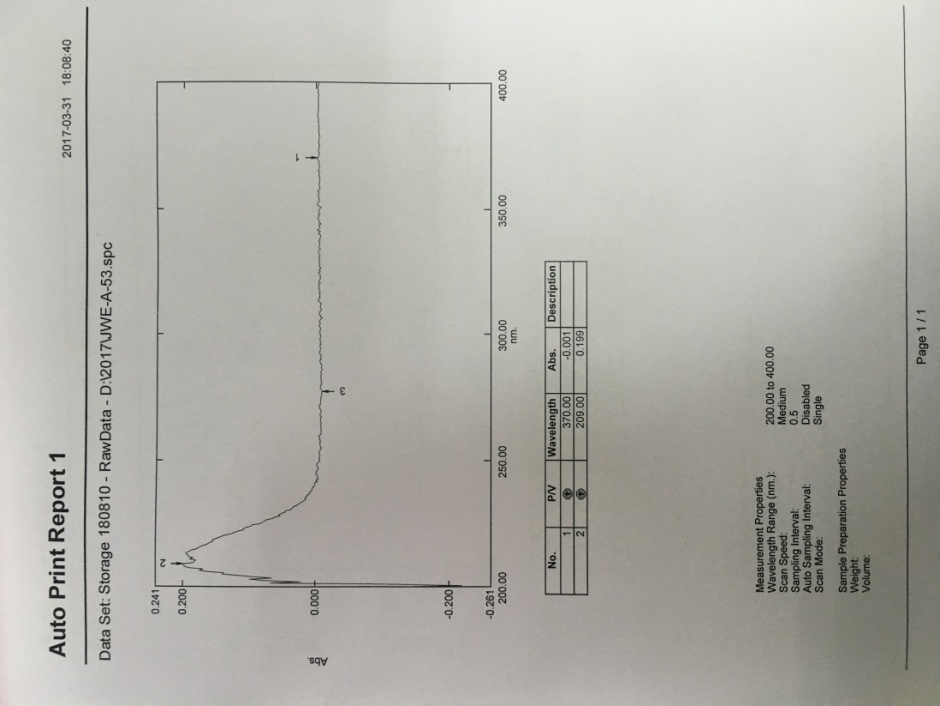


Fig. S4.7 UV spectrum of divarolide G (**4**)


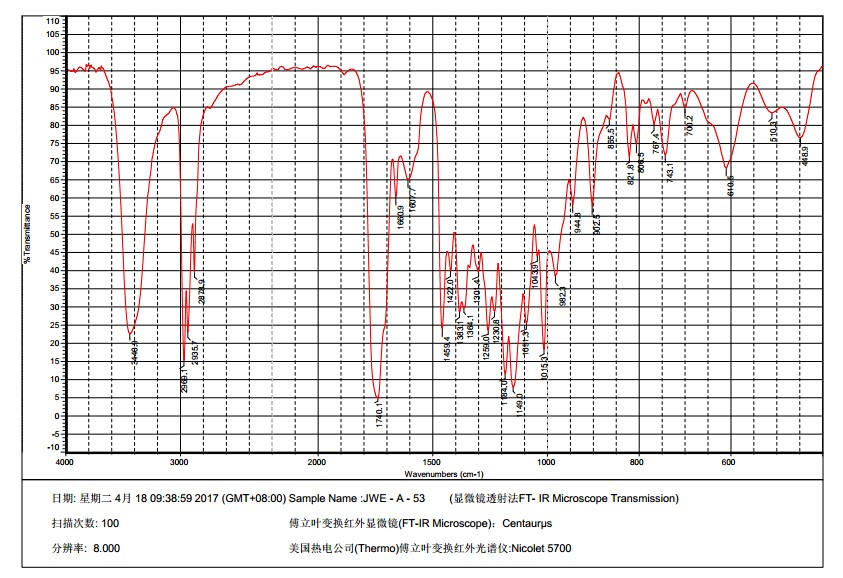


Fig. S4.8 IR spectrum of divarolide G (**4**)


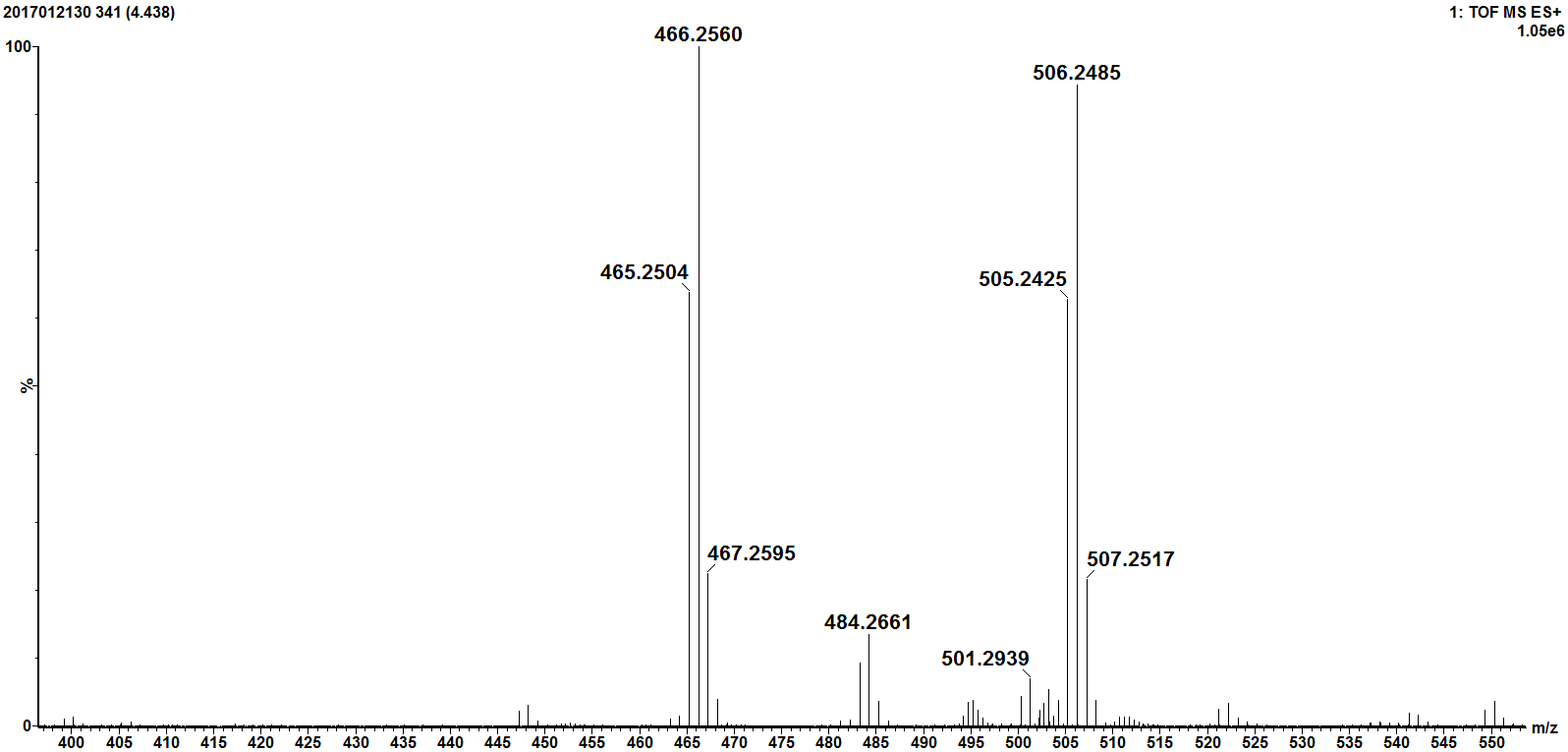


Fig. S4.9 HRESIMS spectrum of divarolide G (**4**)

Fig. S4.10 CD spectrum of divarolide G (**4**)
